# Retraction: Hyper-osmolarity environment-induced oxidative stress injury promotes nucleus pulposus cell senescence in vitro

**DOI:** 10.1042/BSR-2019-1711_RET

**Published:** 2024-08-22

**Keywords:** intervertebral disc, nucleus pulposus, osmolarity, oxidative stress, senescence

This article is one of twenty-five publications being retracted from *Bioscience Reports* at the request of the Editor-in-Chief and the Editorial Board following receipt of a notification from a reader, alerting the Editorial Office to suspected misconduct in three of these articles.

Following this notification, the Editorial Office has investigated the three articles in question, and identified links to a further twenty-two publications with compromised peer review, duplicated images and authorship concerns. The full list of twenty-five publications is as follows:

Li et al, 2017 (DOI: 10.1042/BSR20160582)
Pang et al, 2017 (DOI: 10.1042/BSR20170718)
Han et al, 2017 (DOI: 10.1042/BSR20171319)
Wang et al, 2018 (DOI: 10.1042/BSR20171454)
Zhou et al, 2018 (DOI: 10.1042/BSR20171551)
Shi et al, 2018 (DOI: 10.1042/BSR20180064)
Zhang et al, 2018 (DOI: 10.1042/BSR20171703)
Fang et al, 2018 (DOI: 10.1042/BSR20180018)
Gao et al, 2018 (DOI: 10.1042/BSR20180544)
Jiang et al, 2018 (DOI: 10.1042/BSR20180670)
Jiao et al, 2018 (DOI: 10.1042/BSR20181176)
Fu et al, 2018 (DOI: 10.1042/BSR20181451)
Yu et al, 2018 (DOI: 10.1042/BSR20181530)
Yang et al, 2018 (DOI: 10.1042/BSR20181708)
Jiang et al, 2019 (DOI: 10.1042/BSR20182375)
Gong et al, 2019 (DOI: 10.1042/BSR20182312)
Xu et al, 2019 (DOI: 10.1042/BSR20182462)
Jiang et al, 2019 (DOI: 10.1042/BSR20190043)
Liu et al, 2019 (DOI: 10.1042/BSR20190170)
Zhao et al, 2019 (DOI: 10.1042/BSR20190163)
Li et al, 2019 (DOI: 10.1042/BSR20190126)
Shan et al, 2019 (DOI: 10.1042/BSR20190853)
Xu et al, 2019 (DOI: 10.1042/BSR20191711)
Xie et al, 2020 (DOI: 10.1042/BSR20191726)
Pang et al, 2020 (DOI: 10.1042/BSR20200262)

The authors have been contacted in regard to the retraction, and disagree with the Journal's decision to retract the article. Given the extent of the issues raised, the Editorial Board stand by the decision to retract the article.

